# Influence of substitution patterns on the antimicrobial properties of pyrrole sulfonamide scaffolds

**DOI:** 10.3389/fchem.2026.1726389

**Published:** 2026-02-06

**Authors:** Ioana C. Marinas, Natalia Simionescu, Nicolae D. Andreiu, Ashraf Al-Matarneh, Tudor Pinteala, Mariana C. Chifiriuc, Cristina M. Al-Matarneh

**Affiliations:** 1 Research Institute of the University of Bucharest-ICUB, Bucharest, Romania; 2 SC Deltarom SRL - Centre for Research and Innovative Services in Advanced Biotechnology, Giurgiu, Romania; 3 Center of Advanced Research in Bionanoconjugates and Biopolymers, “Petru Poni” Institute of Macromolecular Chemistry of Romanian Academy, Iasi, Romania; 4 Faculty of Biology, University of Bucharest, Bucharest, Romania; 5 Faculty of Chemistry, Alexandru Ioan Cuza University of Iasi, Iasi, Romania; 6 Department of Orthopedics and Traumatology, Faculty of Medicine, “Grigore T. Popa” University of Medicine and Pharmacy, Iasi, Romania; 7 Department Orthopedics and Traumatology, Clinical Rehabilitation Hospital, Iasi, Romania; 8 Romanian Academy, Bucharest, Romania

**Keywords:** antimicrobial activity, biocompatibility, enzyme inhibition, *m*-sulfonamide, *p*-sulfonamide, pyrrol-2-one

## Abstract

Two series of sulfonamide derivatives featuring a pyrrol-2-one core were synthesized and evaluated for their antimicrobial and anti-virulence features using *Escherichia coli*, *Pseudomonas aeruginosa*, and *Candida albicans* strains, in planktonic and biofilm growth state. Fourteen substituents were introduced on the pyrrole ring, and the sulfonamide group was shifted from *meta*- (Series B) to *para*-position (Series A). *Meta*-substituted sulfonamides generally exhibited stronger antibacterial activity, likely via selective inhibition of microbial *β*-/*γ*-class carbonic anhydrases, while *para*-substituted derivatives demonstrated superior antifungal activity and antibiofilm potential. Also, series A compounds were particularly effective in inhibiting virulence factors, including haemolysin (*S. aureus*), lipase and acidification (*C. albicans*), and lecithinase (*P. aeruginosa*). Structure–activity relationships revealed that *para*-substitution aligns with human CA II, correlated with an enhanced antifungal efficacy, whereas *meta*-substitution favors microbial CA targeting, explaining antibacterial selectivity. Antimicrobial efficacy correlated weakly with lipophilicity and solubility, underscoring species-specific activity. Lipophilicity increased skin permeability but decreased solubility, negatively affecting biocompatibility. However, none of the tested compounds were haemolytic at 1 mg/mL, and all were well tolerated by dermal fibroblasts and keratinocytes at 10 µM. Collectively, these results highlight the dual functionality of these derivatives as selective anti-virulence and antimicrobial agents, while their skin-friendly properties make them promising candidates for the treatment of dermal infections.

## Graphical Abstract

**Figure:**
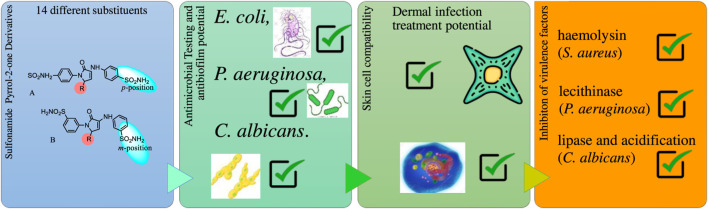

## 1 Introduction

As a foundational class of antimicrobial agents, sulfonamides have significantly contributed to the advancement of modern chemotherapy. Ongoing medicinal chemistry efforts have focused on enhancing their biological activity through the strategic incorporation of heterocyclic scaffolds (Almalki et al., 2022; Krátký, 2024; Krátký et al., 2012). These structural modifications not only improve pharmacokinetic and pharmacodynamic profiles but also broaden the antimicrobial spectrum of sulfonamide derivatives (Diaconu et al., 2020). In particular, heterocycle-sulfonamide hybrids have attracted considerable attention for their potential in dermal applications, including wound healing and infection control. The integration of heterocyclic moieties (such as thiazoles, pyrimidines, quinolines, oxazolones, and pyrroles) into sulfonamide frameworks has led to significant gains in antimicrobial efficacy, including activity against drug-resistant strains such as methicillin-resistant *Staphylococcus aureus* (MRSA). For instance, sulfonamides bearing 5-chloro-2-hydroxybenzaldehyde, thiazole, or pyrimidine groups have demonstrated potent antibacterial activity against both Gram-positive and Gram-negative bacteria, as well as against mycobacteria. In addition to improved antimicrobial potency, some of these hybrid structures exhibit enhanced pharmacological properties, including anti-inflammatory, antioxidant, anticancer, and antiviral activities (Almalki et al., 2022; Krátký, 2024; Krátký et al., 2012; Moskalik, 2022).

While sulfonamides are traditionally recognized as bacteriostatic agents *via* inhibition of folic acid biosynthesis, recent derivatives have demonstrated additional modes of action, such as interference with *quorum sensing* and suppression of biofilm formation (Almalki et al., 2022; Krátký, 2024). This multi-targeted activity is particularly valuable in the context of antimicrobial resistance (AMR) (Garudachari et al., 2012), a mounting global health threat often described as a “slow tsunami” capable of rendering current antibiotics ineffective (D’Agostino et al., 2022). The need for novel chemotypes with broad-spectrum efficacy and resistance-evasion potential has become increasingly urgent (Naaz et al., 2018), especially in the management of nosocomial infections and chronic wounds (Kumar Verma et al., 2020).

In this context, sulfonamide-based scaffolds have shown promise in tissue engineering applications. Their integration into dermal matrices-fabricated *via* electrospinning, 3D printing, or other advanced techniques, enables sustained antimicrobial delivery directly to the site of infection. Combining sulfonamides with other bioactive agents or nanomaterials has further enhanced their utility in preventing microbial colonization and promoting tissue regeneration (Serrano-Aroca et al., 2022).

The medicinal relevance of the sulfonyl and sulfonamide functional groups extends far beyond antibacterial activity (Meanwell, 2011; Wang et al., 2010). These moieties are integral to a wide range of bioactive molecules (Majumdar and Mondal, 2011), exhibiting properties such as antifungal (Lal et al., 2013), diuretic (Allen and Lee, 1973), carbonic anhydrase inhibition (Angeli et al., 2022) and cytotoxicity against tumoral cells (Konda et al., 2015). Numerous sulfonamide-bearing heterocycles-including quinazolinones, benzimidazoles, and thiazoles-have been successfully developed and demonstrate excellent efficacy against both standard and multidrug-resistant pathogens (Bano et al., 2011).

**FIGURE 1:**
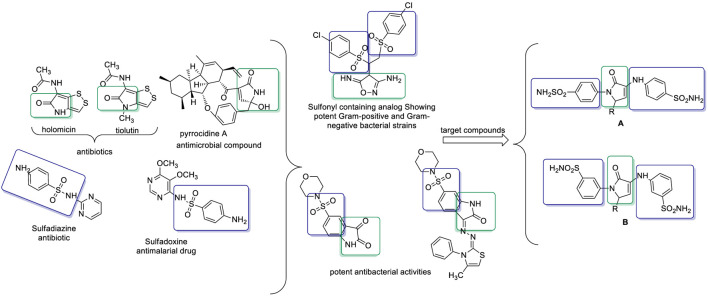
Design for the pyrrol-sulfonamide bioactive compounds.

Pyrrole-based compounds, particularly pyrrol-2-one derivatives, constitute a vital class of heterocycles with broad pharmacological potential (Amariucai-Mantu et al., 2023). Found in a variety of natural products, such as pyrrocidine A, holomycin, and thiolutin (Figure 1) (Acid et al., 2018), these compounds exhibit antibacterial (Demi et al., 1999), cytotoxic (Geng et al., 2015), and antioxidant properties (Kim et al., 2007), and serve as inhibitors of key enzymes (Alizadeh et al., 2022; Alp et al., 2010) and protein–protein interactions (Reddy et al., 2011). Their structural versatility and synthetic accessibility make them attractive candidates for further development in antimicrobial drug discovery.

Despite advances in rational drug design, including *in silico* modeling and high-throughput screening, the process of identifying effective antimicrobial agents continues to rely heavily on experimental validation and structure–activity relationship (SAR) analysis (Musiol, 2017). The development of sulfonamide–heterocycle hybrids represent a rational yet innovation-driven approach to expanding the therapeutic arsenal against resistant and emerging pathogens. Continued exploration of these hybrid systems is essential to meet the growing demand for effective, safe, and multifunctional antimicrobial agents.

Taking all this data into account, the aim of our current study was to compare the antimicrobial activity of novel two position substitution sulfonamide–pyrrole scaffolds. This work represents a continuation of our ongoing research on N-heterocyclic systems (Al Matarneh et al., 2016a; Al Matarneh et al., 2016b; Al Matarneh et al., 2019; Al-Matarneh et al., 2021) and aligns with our broader interest in physiologically active compounds, particularly pyrrol-2-one derivatives (Al-Matarneh et al., 2023; Al-Matarneh et al., 2024; Al-Matarneh et al., 2025).

## 2 Results and discussion

### 2.1 Synthesis and characterization

Heterocyclic rings, particularly those containing nitrogen, are fundamental components in many modern antimicrobial agents. The incorporation of nitrogen atoms enhances lipophilicity and facilitates hydrogen bonding, contributing to improved pharmacological and pharmacokinetic profiles while often reducing toxicity. As a result, nitrogen-containing heterocycles frequently serve as core structural motifs in both natural and synthetic antimicrobial compounds.

**FIGURE 2:**
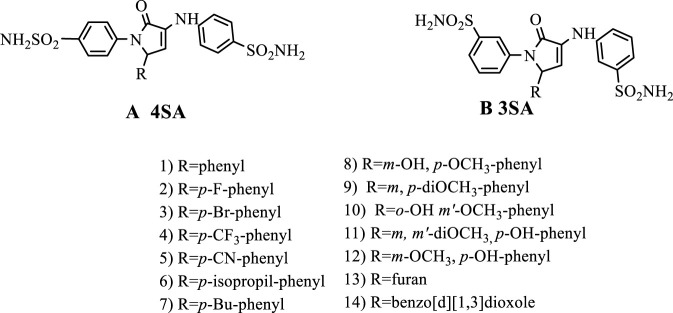
General formulas for the compared compounds.

Thus, we have designed two series of pyrrole-sulphonamide derivatives (Figure 2: compounds **1-14 A** and **1-14 B)** in order to have them tested against microbial stains such as *E. coli*, *P. aeruginosa* and *C. albicans*. The derivatives have been recently synthesized in our group (Al-Matarneh et al., 2024; Al-Matarneh et al., 2025) using sulfonamide *p-* or *m*-aniline substituted, different aldehydes and pyruvic acid in ethanolic media. All compounds have been fully characterized using spectral methods (FTIR, MS, NMR).

This study aimed to compare the biological activities of sulfonamide derivatives based on the position of the sulfonamide group on the phenyl ring (*meta* or *para*). By incorporating a diverse set of electron-donating and electron-withdrawing substituents, as well as varying the number of substituents, we conducted a comprehensive evaluation. The resulting data provide a valuable foundation for identifying promising candidates with enhanced antimicrobial potential.

### 2.2 Antimicrobial activity

Pyrrole analogs have diverse therapeutic applications, such as fungicides, antibiotics, anti-inflammatory drugs, cholesterol-reducing drugs, and antitumor agents (Maheshwari and Bandyopadhyay, 2021). The combination of different pharmacophores in a pyrrole and pyrrolidine ring system has led to more active compounds (Ahmad et al., 2018; Jeelan Basha et al., 2022). Pyrrole antibiotics are naturally occurring antibiotics that have a nitrogen pyrrole nucleus that is chemically stable and reactive. The antifungal efficacy of pyrrole against *C. albicans* and *Trichophyton mentagophytes* strains was shown to be diminished after N-alkylation (Jahnavi, 2023). On the other hand, sulfonamides are still widely used bacteriostatic drugs around the world, owing to their inexpensive cost, minimal toxicity, and excellent efficacy against common bacterial infections (Jubeh et al., 2020; Nunes et al., 2020).

Considering the urgent need for novel anti-infective medications, the synthesized compounds were evaluated *in vitro* for antimicrobial activity against *S. aureus* ATCC 25923, *P. aeruginosa* ATCC 27853, and *C. albicans* ATCC 10231 strains using quantitative tests. Because of their antibiotic resistance, *S. aureus* and *P. aeruginosa* infections remain unresolved challenges, ranking among the most feared opportunistic and nosocomial bacterial agents. Both organisms have the potential to cause serious infections (Almuhayawi et al., 2023; Guo et al., 2023).

*Candida* spp. are opportunistic fungal pathogens that can cause a variety of diseases in humans, including mucosal candidiasis and invasive candidiasis. Candidemia, the most severe form of invasive candidiasis, is becoming increasingly common in tertiary care hospitals around the world, causes significant morbidity and mortality (Reda et al., 2023).

**FIGURE 3:**
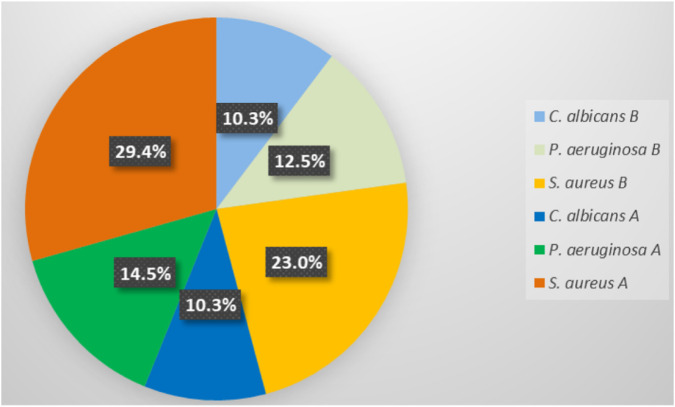
The percentage share of antimicrobial activities given by the MIC values for A and B series.

The minimum inhibitory concentration (MIC) values against the tested microorganisms are reported in Table 1. The range of the MIC values for the majority of the tested compounds was from 0.625 to 10 mg/mL. The compound 9B had significant antibacterial activity against *S. aureus* With a few exceptions (2A vs. 2B, 3A vs. 3B, 10A vs. 10B, 12A vs. 12B and 14A vs. 14B), the sulfonamide groups in the *meta* position (B series) amplify the antibacterial activity compared to that in the *para* position (A series) (Figure 3). The overall difference between the two series was 6.36%. The order of antibacterial activity on the tested Gram-positive strain for series A was 2A > 3A = 12A = 14A > **1A** = 4A = 5A = 10A = 13A > 9A = 8A = 11A = 7A = 6A, while for series B was 9B > 4B = 5B = 6B = 7B > 2B = 3B = 12B = 13B = 14B = **1B** > 8B = 10B = 11B. In terms of bactericidal activity, it was found that only compounds 3B and 4B had an effect on the *S. aureus* strain at a concentration of 5 mg/mL.

**TABLE 1:** Minimum inhibitory concentrations (MIC), minimum microbicidal concentration (MMC) and minimum biofilm eradication concentration (MBEC) values.

No.	Code	*S. aureus*			*P. aeruginosa*			*C. albicans*		
		MIC (mg/mL)	MMC (mg/mL)	MBEC (mg/mL)	MIC (mg/mL)	MMC (mg/mL)	MBEC (mg/mL)	MIC (mg/mL)	MMC (mg/mL)	MBEC (mg/mL)
1	1A	5	10	5	2.5	5	0.156	2.5	5	0.313
2	2A	1.25	10	1.25	1.25	5	1.25	0.313	2.5	0.156
3	3A	2.5	10	0.625	1.25	5	0.156	1.25	5	0.156
4	4A	5	>10	0.625	1.25	5	0.156	2.5	5	0.156
5	5A	5	>10	0.156	1.25	2.5	0.156	5	5	0.313
6	6A	10	>10	1.25	2.5	5	1.25	0.625	5	0.313
7	7A	10	10	0.625	1.25	5	2.5	0.313	2.5	0.156
8	8A	10	>10	10	5	5	2.5	0.313	5	0.156
9	9A	10	10	1.25	10	10	0.156	0.313	5	0.156
10	10A	5	10	0.625	5	5	0.156	2.5	2.5	2.5
11	11A	10	10	5	2.5	5	0.156	10	10	2.5
12	12A	2.5	10	0.313	2.5	5	0.156	2.5	2.5	0.313
13	13A	5	10	2.5	2.5	5	2.5	0.625	2.5	0.625
14	14A	2.5	10	0.625	2.5	5	2.5	0.625	10	0.625
15	1B	5	10	0.313	2.5	5	0.156	1.25	2.5	0.156
16	2B	5	10	5	2.5	5	2.5	2.5	2.5	0.156
17	3B	5	5	1.25	5	5	0.625	1.25	2.5	0.156
18	4B	1.25	5	2.5	1.25	5	0.156	1.25	2.5	0.156
19	5B	1.25	10	0.156	1.25	5	0.156	0.625	5	0.313
20	6B	1.25	10	2.5	1.25	1.25	0.156	2.5	2.5	0.625
21	7B	1.25	10	5	1.25	2.5	2.5	2.5	2.5	2.5
22	8B	10	>10	10	2.5	5	2.5	2.5	2.5	0.313
23	9B	0.625	>10	0.625	0.625	5	0.156	0.625	2.5	0.156
24	10B	10	10	5	2.5	2.5	0.156	2.5	2.5	2.5
25	11B	10	10	2.5	2.5	5	0.156	0.625	2.5	0.156
26	12B	5	10	5	5	5	0.313	5	5	0.156
27	13B	5	>10	5	2.5	>10	2.5	5	5	0.156
28	14B	5	>10	5	5	5	0.313	1.25	2.5	1.25
​	G^a^	0.0044	0.0175	0.0044	0.0088	0.0088	0.0088	-	-	-
​	K^a^	-	-	-	-	-	-	0.0175	0.07	0.0175
​	DMSO	5	10	2.5	2.5	5	2.5	2.5	5	2.5

^a^ G – Gentamycin, K - Ketoconazole.

In the case of the *P. aeruginosa* strain, the MIC values of the newly synthesized compounds were between 0.625 - 5 mg/mL. From Table 1, the compound 9B being again the most active. For the comparison between series A and B (Figure 3), it was observed that series B has a better antibacterial activity against the tested Gram-negative strain than series A with a few exceptions: 2A vs. 2B, 3A vs. 3B, 12A vs. 12B and 14A vs. 14B. The global difference between the two series was 1.97%. The order of antimicrobial activity on *P. aeruginosa* strain for series A is 2A = 3A = 4A = 5A = 7A > **1A** = 11A = 12A = 14A = 13A = 6A > 10A = 8A > 9A, and for series B: 9B > 4B = 5B = 6B = 7B > **1B** = 2B = 8B = 10B = 11B = 13B > 3B = 12B = 14B. From the point of view of microbicidal activity, it was observed that the *P. aeruginosa* strain is sensitive to compounds 6B, 7B, 10B and 5A.

In the case of the *C. albicans* strain, the MIC values were between 0.313 - 10 mg/mL. From Table 1, it can be seen that the variants 2A, 9A, 8A and 7A presented the best activity. The global difference between the A and B series was very low (approx. 0.0001%), indicating a similar antifungal activity of the two series (Figure 3). The order of antimicrobial activity on *C. albicans* for series A was 9A = 2A = 7A = 8A > 13A = 14A = 6A > 3A > **1A** = 10A = 12A = 4A > 5A > 11A, and for series B: 5B = 9B =11B > **1B** = 3B = 4B = 14B > 8B = 2B = 10B = 6B = 7B > 13B = 12B. It was observed that the *C. albicans* strain was sensitive to the compounds 2A, 7A, 8A, 9A, 6A, 13A, 14A, 5B, 9B and 11B. For comparison, the corresponding values for the reference compounds gentamicin and ketoconazole are also presented in Table 1. However, the analysis of the discussions focused on the relationship between the chemical structure and biological activity of the two series of 14 synthesized derivatives, with the main goal being to identify internal trends that can guide future syntheses of compounds with improved antimicrobial potential.

**FIGURE 4:**
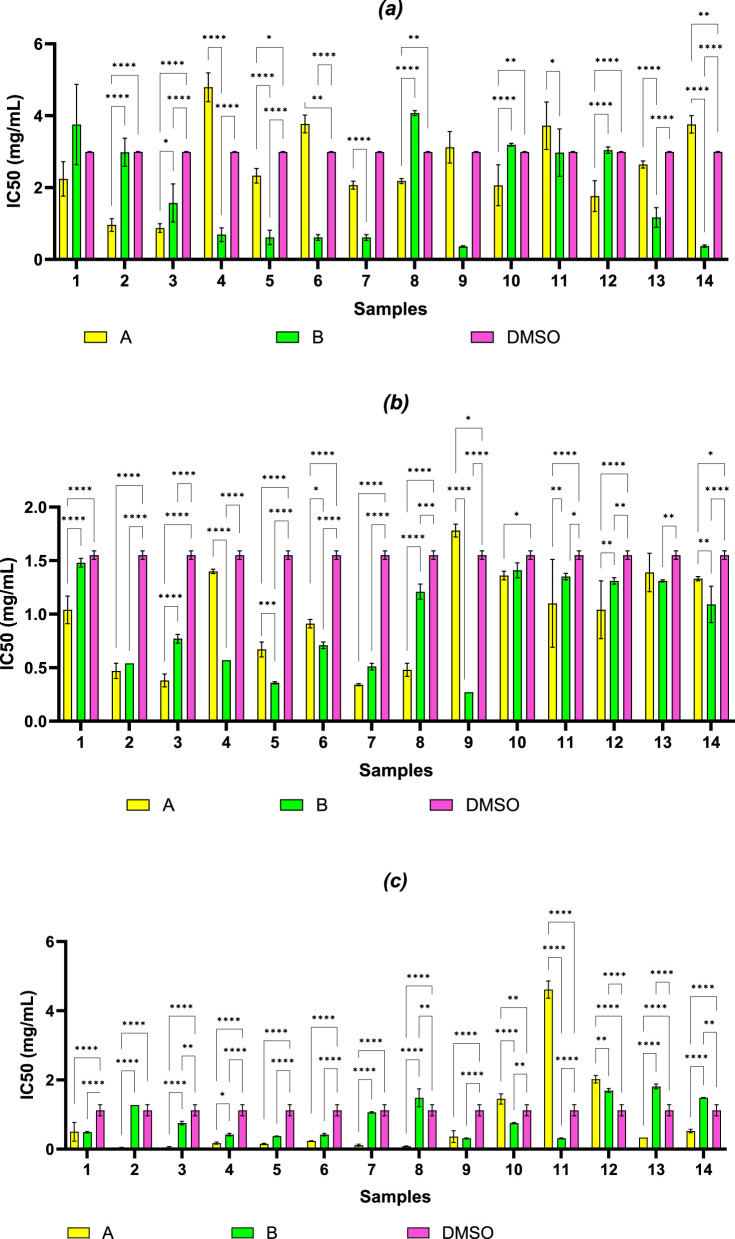
IC50 values of the tested compounds against: **(a)** *S. aureus*; **(b)** *P. aeruginosa* and **(c)** *C. albicans.* Statistical analysis was performed using the two-way ANOVA method (Tukey’s multiple comparisons test), and significance was expressed as follows: *p < 0.05, **p < 0.01, ***p < 0.001, ****p < 0.0001 (n = 3).

The IC50 values for each compound against the tested microorganisms were summarized in Figure 4a for *S. aureus*, Figure 4b for *P. aeruginosa* and Figure 4c for *C. albicans*. The IC_50_ values were analyzed using a two-way ANOVA to evaluate the effects of compound series. Data distribution was assessed using the Shapiro–Wilk test, and variance homogeneity was confirmed (p > 0.05). Although the data did not strictly follow a normal distribution (p < 0.001), ANOVA was applied given the homogeneity of variances and the robustness of this test under slight deviations from normality. The compounds 3A, 2A, 4B, 5B, 6B, 7B, 9B, 13B and 14B exhibited the highest activity against *S. aureus* as revealed from the IC50 values that were between 0.36 and 1.17 mg/mL. From the IC50 values, in the case of *P. aeruginosa* it was observed that compounds 9B (p < 0.0001 compared to DMSO and compared to 9A) and 5B (p < 0.0001 compared to DMSO and p < 0.01 compared to 5A) and 4B (p < 0.0001 both against DMSO and against 4A) were the most active, with computed values ​​of 0.27–0.57 mg/mL. In the case of *C. albicans*, the IC50 values were significantly lower for the A series, the most active compounds being 2A, 3A, 4A, 5A, 6A, 7A, 8A, 9A, 13A and 14A, while from the B series, the compounds with significantly lower IC50 values compared to DMSO were 1B, 3B, 4B, 5B, 6B, 9B, 10B and 11B (p < 0.01).

The MBEC values obtained for the microbial strains tested in this study demonstrate distinct variations in antibiofilm activity between the derivatives from the two series. Improved mesomeric electronic conjugation, increased planarity of the aromatic core, and alignment of dipole vectors facilitating diffusion through the biofilm matrix can be considered possible antibiofilm mechanisms due to the *para*-location of the -SO_2_NH_2_ group in series A (Pinto et al., 2020; Saxena et al., 2023; Shaw et al., 2014; Weliwatte et al., 2021; Wheeler and Bloom, 2014). In contrast, the *meta* position in the B series limits these effects, probably providing less favourable orientations for the sulfonamide group’s exposure to the environment. Thus, compounds in series A more frequently showed MBEC values lower than MIC, suggesting a specific biofilm disruption mechanism, and MBEC values lower than the control solvent (DMSO), confirming that the observed activity is not solely due to the solvent effect.

This structural difference between series A and B was reflected in the antimicrobial activity against all three tested microorganisms, but it was most pronounced in *P. aeruginosa*, where the additional barrier represented by the outer membrane and biofilm matrix penalises compounds that are less planar or have exposed polar groups (Ghosh et al., 2023; Kotowska et al., 2023; Lam et al., 2020). In the case of *S. aureus* and *C. albicans*, the advantage of the A series remains visible, but the difference between the series is lower compared to that of the Gram-negative *bacillus*.

Analysing the effect of substituents, several clear trends emerge. Halogenated and lipophilic electron-withdrawing substituents (Figure 2: *p*-F-phenyl (2), *p*-Br-phenyl (3), *p*-CF_3_ (4), *p*-CN (5)) frequently associate with low MBECs, often even below the MIC value, especially in series A, where the *para* vector maximises their effect of increasing lipophilicity and promoting hydrophobic and π-π interactions (Faleye et al., 2024; Mengelers et al., 1997). Alkyl substituents (Figure 2: *p*-*Iso*Pr-phenyl (6) and *p*-^t^Bu-phenyl (7)) are also beneficial, enhancing hydrophobic anchoring in the biofilm matrix and maintaining low MBEC values against *S. aureus* and *C. albicans*. Strong donor substituents (OH, OCH_3_, and di-OCH_3_ (9, 10, 11, 12)) on the other hand, enhance polarity and penalise transport through the biofilm, particularly in *P. aeruginosa*. Since certain substitutions (*meta*- or *para*-hydroxy and methoxy) can still provide MBECs below MIC, two potential alternative mechanisms involved are ion chelation from the matrix or loss of biofilm cohesiveness (Ivanova et al., 2023; Rath et al., 2025; Tartari et al., 2025; Zhou K. et al., 2025). While the heteroaromatic radical (benzodioxole (14)) benefited from the possibility of aromatic stacking, its activity was constrained by its enhanced polarity.

Taken together, our data reveal that Series A derivatives provide a distinct advantage for the antibiofilm effect due to the *para*-orientation of the -SO_2_NH_2_ group. This effect was demonstrated by the lower MBEC values and a and very often, even lower than the corresponding MIC values. Halogens, CF_3_, CN, and alkyls are the most consistent promoters of antibiofilm activity, while polar substituents offer specific benefits but tend to decrease performance against microorganisms with high permeability barriers (Faleye et al., 2024; Hanot et al., 2025; Wang S. et al., 2021). These correlations indicate that the *para* position of the sulfonamide was optimal for diffusion and interactions with the biofilm, and the choice of substituents should be guided by a balance between lipophilicity and polarity, with a slight preference for hydrophobic radicals, especially when the target is *P. aeruginosa* strain.

Similar virulence factors are used by pathogens, including *S. aureus* and *P. aeruginosa,* to colonise tissues, create biofilms, and slow the healing process in chronic wound infections. Polymicrobial colonisation of wounds involves both opportunistic yeasts, mainly *C. albicans*, and bacteria, such as *S. aureus*, *P. aeruginosa*, and Enterobacteriaceae (Bowler et al., 2001; Mariani and Galvan, 2023). Among the most important soluble toxins, haemolysins cause the lysis of erythrocytes and other host cells, releasing iron and other nutrients (Berube and Wardenburg, 2013; Divyakolu et al., 2019; Shumba et al., 2019). In chronic wounds, it maintains inflammation and necrosis (Uberoi et al., 2024; Yang et al., 2024). Inhibiting haemolysins limits tissue destruction and reduces metabolic resources for bacteria. Among haemolysins, lecithinases (phospholipase C) attack the phospholipids of cell membranes, causing necrosis, local haemorrhage, and stimulating the inflammatory response (Karasawa et al., 2003; Singh et al., 2023; Titball, 1993). In the particular context of chronic wounds, lecithinase activity amplifies destruction and delays healing. Blocking this activity reduces the severity of injuries and supports regeneration (Muttiah and Hanafiah, 2025; Zhou L. et al., 2025). Lipases degrade lipids in tissues and sebum, affecting the skin barrier and generating energy sources that favor the multiplication of bacterial pathogens (Yao et al., 2021), (Cheng et al., 2025). In *C. albicans*, phospholipases are essential for tissue invasion and host colonisation. Their suppression limits both the penetration of microbial cells and the inflammatory response (da Silva Dan et al., 2016). Lipases also contribute to the maintenance of inflammation and additional tissue damage in chronic wounds. Thus, their inhibition could reduce invasion and support the restoration of the tissue barrier (Bender and Flieger, 2010; Chen and Alonzo, 2019). DNases activity promotes microbial dispersion and penetration into host tissues, regardless of the possibility that it might render the biofilm unstable by depolymerisation of the extracellular DNA present in the biofilm matrix (Rath et al., 2025). Thus, DNase inhibition could act as a trap of bacteria inside biofilms, slowing tissue invasion, thus limiting the lesions progression and making bacterial infections more amenable to therapy (Garcia Gonzalez and Hernandez, 2022). Metabolic adaptability aids bacterial survival in the hypoxic and nutrient-poor environment of chronic wounds. For example, the capacity of intestinal bacteria and other microorganisms to metabolise complex glycosides is also due to the presence of aesculin hydrolases (Wang C. et al., 2021). Restricting this action lowers microbial persistence and metabolic adaptation (Acierno et al., 2025; Onyango and Alreshidi, 2018). Therefore, inhibiting the production of these virulence factors and metabolic features has major implications for controlling the clinical evolution of infected chronic wounds. Inhibiting lipases restricts the invasion and degradation of the lipid barrier (Zhou H. et al., 2025), inhibiting haemolysin and lecithinase reduces the aggressiveness of microorganisms towards tissues and inflammation (Fik et al., 2005), and modifying DNase disrupts biofilm dispersion and metabolic adaptability (Deng et al., 2024). Sulfonamide derivatives that affect these pathways can therefore be considered both conventional antimicrobials and anti-virulence agents, which can help heal chronic wounds by reducing the pathogen’s impact.

From Table 2, it can be observed that, in the case of *S. aureus* strain, most compounds (excepting 7B) block totally or partially (5B, 50%) the haemolysin secretion, at 0.625 mg/mL. The *para* orientation of the sulfonamide favoured the suppression of haemolysin secretion, while in the *meta* position, a loss of efficacy was observed for some substituents (*p*-CN-phenyl and *p*-^t^Bu-phenyl). A possible explanation would be the more efficient interaction of compounds from the A series with the membrane/regulatory cascades (Agr/Sae), leading to a decrease in the secretion of haemolytic toxins (Kong et al., 2016). Some compounds significantly stimulated DNase activity compared to the control (1A = 150.00 ± 0.00%, 2A = 112.5 ± 17.68%, 8A = 150.00 ± 0.00%, 9A = 112.5 ± 17.68%), while others have totally (3A and 10A) or partially (4A, 5A, 6A, 11A, 12A, 3B, 4B, 7B, 10B, 11B, 14B) inhibited this activity. None of the compounds in series B stimulate enzymatic activity, and some have significantly inhibited DNase activity (3B, 7B, 10B, 14B). According to Table 2, it can be observed that the solvent used also had an inhibitory effect, but the difference was insignificant compared to the strain control (p > 0.05). Many compounds reduced aesculin hydrolase activity to moderate levels (33%–83%: 1A, 2A, 3A, 4A, 5A, 6A, 8A, 9A, 10A, 11A, 1B, 2B, 3B, 4B, 5B, 6B, 8B, 10B, 11B, 12B), and a single compound completely inhibited it (7B). A single compound led to stimulation (14A), with a value of 116% ± 23.57%, but the difference was not statistically significant compared to the strain control (p > 0.05). Comparing the two series of derivatives, series A has more effectively reduced this enzymatic pathway than the compounds in series B. Sulfonamide derivatives appear to stimulate lipase secretion in *S. aureus*, possibly through compensatory mechanisms, which could represent a bacterial adaptation response. However, two compounds from the B series managed to block lipase secretion, i.e., 13B and 14B. In the case of lecithinase, a stimulation of this enzyme was induced by a subset of compounds from series A (1A, 3A) and B (1B, 3B, 7B). In conclusion, series A (*para*-SO_2_NH_2_) exhibits a more favourable anti-virulence profile sustained by the ability of these compounds to consistently inhibit haemolysin, lecithinase and aesculin hydrolase, although it may increase DNase, this effect being potentially pro-dispersal (Deng et al., 2024). On the other hand, the wound secretions fluidization could facilitate the access of antibiotics and immune cells to the site of infection. The *S. aureus* strain under investigation did neither produce gelatinase, nor cause pH changes. Overall, sulfonamide derivatives selectively modulate the secretion of virulence factors produced by the *S. aureus* strain, the effect depending strongly on the nature of the substituent.

**TABLE 2:** Effect of sulfonamide derivatives on the secretion of enzymatic virulence factors (*S. aureus*). The colours used indicate: green – complete inhibition; yellow – reduced enzymatic activity; orange – activity similar to the strain control; red – stimulation of enzymatic activity. Statistical analysis was performed using Brown-Forsythe and Welch ANOVA test followed by Dunnett’s multiple comparisons test (n = 3).

Compound/Reference tested strain	Hemolysin (%)			DN-ase (%)			Aesculin hydrolyse (%)			Lipase (%)			Lecitinase (%)		
	Mean	Std. dev.	p-value	Mean	Std. dev.	p-value	Mean	Std. dev.	p-value	Mean	Std. dev.	p-value	Mean	Std. dev.	p-value
*S. aureus contro*	100	0	-	100	0	-	100	0	-	100	0	-	100	0	-
1A	0	0	<0.0001	150	0	0.0261	75	11.79	>0.05	216.67	23.57	<0.0001	150	0	<0.0001
2A	0	0	<0.0001	112.5	17.68	>0.05	83.33	23.57	>0.05	116.67	23.57	>0.05	100	0	>0.05
3A	0	0	<0.0001	0	0	<0.0001	75	11.79	>0.05	133.33	0	>0.05	112.5	17.68	>0.05
4A	0	0	<0.0001	62.5	17.68	>0.05	66.67	0	>0.05	83.33	23.57	>0.05	87.5	17.68	>0.05
5A	0	0	<0.0001	75	35.36	>0.05	66.67	0	>0.05	133.33	0	>0.05	100	0	>0.05
6A	0	0	<0.0001	75	35.36	>0.05	50	23.57	0.0105	66.67	0	>0.05	62.5	17.68	0.0046
7A	0	0	<0.0001	100	0	>0.05	100	0	>0.05	83.33	23.57	>0.05	100	0	>0.05
8A	0	0	<0.0001	150	0	0.0261	66.67	0	>0.05	50	23.57	0.0285	62.5	17.68	0.0046
9A	0	0	<0.0001	112.5	17.68	>0.05	41.67	11.79	0.0016	66.67	0	>0.05	75	0	>0.05
10A	0	0	<0.0001	0	0	<0.0001	83.33	23.57	>0.05	250	23.57	>0.05	75	0	>0.05
11A	0	0	<0.0001	62.5	17.68	>0.05	83.33	23.57	>0.05	150	23.57	0.0285	100	0	>0.05
12A	0	0	<0.0001	75	35.36	>0.05	100	0	>0.05	216.67	23.57	<0.0001	100	0	>0.05
13A	0	0	<0.0001	75	35.36	>0.05	100	0	>0.05	33.33	0	0.0010	100	0	>0.05
14A	0	0	<0.0001	100	0	>0.05	116.67	23.57	>0.05	116.67	23.57	>0.05	50	0	<0.0001
1B	0	0	<0.0001	100	0	>0.05	83.33	23.57	>0.05	116.67	23.57	>0.05	125	0	>0.05
2B	0	0	<0.0001	100	0	>0.05	83.33	23.57	>0.05	66.67	0	>0.05	100	0	>0.05
3B	0	0	<0.0001	25	0	0.0001	66.67	0	>0.05	183.33	23.57	<0.0001	150	0	<0.0001
4B	0	0	<0.0001	75	35.36	>0.05	33.33	0	0.0002	183.33	23.57	<0.0001	62.5	17.68	0.0046
5B	50	0	<0.0001	100	0	>0.05	66.67	0	>0.05	116.67	23.57	>0.05	75	35.36	>0.05
6B	0	0	<0.0001	100	0	>0.05	66.67	0	>0.05	133.33	0	>0.05	87.5	17.68	>0.05
7B	100	0	>0.05	50	0	0.0261	0	0	<0.0001	183.33	23.57	<0.0001	137.5	17.68	0.0046
8B	0	0	<0.0001	100	0	>0.05	66.67	0	>0.05	56.67	14.14	0.0876	75	0	>0.05
9B	0	0	<0.0001	100	0	>0.05	66.67	0	>0.05	250	23.57	<0.0001	100	0	>0.05
10B	0	0	<0.0001	25	0	0.0001	83.33	23.57	>0.05	283.33	23.57	<0.0001	100	0	>0.05
11B	0	0	<0.0001	75	35.36	>0.05	83.33	23.57	>0.05	266.67	0	<0.0001	62.5	17.68	0.0046
12B	0	0	<0.0001	100	0	>0.05	83.33	23.57	>0.05	183.33	23.57	<0.0001	100	0	>0.05
13B	0	0	<0.0001	100	0	>0.05	83.33	23.57	>0.05	0	0	<0.0001	100	0	>0.05
14B	0	0	<0.0001	50	0	0.0261	91.67	35.36	>0.05	0	0	<0.0001	87.5	17.68	>0.05
DMSO	56.25	8.84	<0.0001	75	35.36	>0.05	83.33	23.57	>0.05	116.67	23.57	>0.05	100	0	>0.05

**FIGURE 5:**
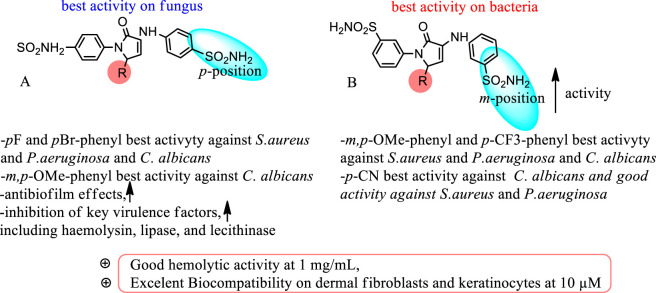
SAR diagram of the active compounds.

In case of *P. aeruginosa* strain, it did not constitutively secrete DNase and did not exhibit aesculin hydrolysis activity. From Table 3, it can be observed that in the case of compounds 4A-4B and 5A-5B, regardless of the base structure, the presence of *p*-CF3-phenyl and *p*-CN-phenyl radicals led to an intensification of haemolysis. This effect was also observed for compounds 6A (*p*-isopropyl-phenyl), 9A (*m, p*-di OCH3-phenyl), and 2B (*p*-F-phenyl), which do not have common radicals. However, 5 compounds from the series A (1A, 2A, 10A, 11A, 12A), and 4 from series B (1B, 10B, 11B, 12B) have completely inhibited the haemolysin production. In the medium used for acid production evaluation, the strain caused an alkaline reaction (red) instead of acidification (yellow) (Figure 5a). This indicates the absence of carbohydrate fermentation and the preferential use of peptones or amino acids, a process characteristic of non-fermenting species such as *P. aeruginosa* (Geremia et al., 2025). In biofilms from chronic wounds, the metabolism of *P. aeruginosa* could contribute to the alkalisation of the local microenvironment. A higher pH can inactivate some host enzymes involved in healing (e.g., matrix proteins, growth factors), can affect the function of neutrophils and macrophages, reducing the effectiveness of the immune response, and can favour bacterial survival by decreasing the effectiveness of some antibiotics that work better in an acidic environment (e.g., aminoglycosides) (Jones et al., 2015; Maurin and Raoult, 2001). According to Table 3, a decrease in pH was observed in most cases compared to the parent compound, with the exception of compounds 12 from both series (*m*-OCH3, *p*-OH-phenyl), for which the basicity increased. Compounds 1A-B, 2A-B, and 3A-B significantly reduced the basicity of the medium compared to the stem control (p < 0.0001). A similar effect was also observed for the solvent used (DMSO). Gelatinase was frequently strongly stimulated (1A, 2A, 3A, 5A, 6A, 7A, 8A, 9A, 10A, 11A, 12A, 1B, 2B, 3B, 4B, 5B, 6B, 7B, 8B, 9B, 10B, 11B, 12B), and for the variants where partial inhibition was observed, this was not statistically significant compared to the strain control (p > 0.05). These results are consistent with other studies showing that most sulfonamides increase the secretion of gelatinase, a proteolytic enzyme involved in the degradation of the extracellular matrix and tissue invasion (Björklund and Koivunen, 2005). Lipase activity was significantly reduced by the compounds 10A, 11A, 12A, 4B, 10B, 11B and 12B, bearing the *o*-OH *m'*-OCH3-phenyl (10), *m, m'*-diOCH3 *p*-OH-phenyl (11), and *m*-OCH3 *p*-OH-phenyl radicals, regardless of the basic structure. Most compounds have significantly inhibited the lecithinase activity (p < 0.0001) compared to the strain control (yellow in Table 3, 1A, 3A, 5A, 7A, 9A, 12A, 13A, 14A, 1-8B, 10-14B), with a number of compounds in the B series having partially inhibited the secretion of this enzyme. Inhibition is a positive point for potential therapeutic use, reducing associated necrosis and inflammation (Schmiel and Miller, 1999).

**TABLE 3:** Effect of sulfonamide derivatives on the secretion of enzymatic virulence factors (*P. aeruginosa*). The colours used indicate: green – complete inhibition; yellow – reduced enzymatic activity; orange – activity similar to the strain control; red – stimulation of enzymatic activity. Statistical analysis was performed using Brown-Forsythe and Welch ANOVA test followed by Dunnett’s multiple comparisons test (n = 3).

Compound/Reference tested strain	Hemolysin			Organic acidity/Alkalinity			Gelatinase			Lipase			Lecitinase		
	Mean	Std. dev.	p-value	Mean	Std. dev.	p-value	Mean	Std. dev.	p-value	Mean	Std. dev.	p-value	Mean	Std. dev.	p-value
*P. aeruginosa*	100	15.71	-	100	5.24	-	100	12.86	-	100	0	-	100	0	-
1A	0	0	<0.0001	51.85	0.00	<0.0001	150.00	6.43	>0.05	100.00	0.00	>0.05	50.00	0.00	<0.0001
2A	0	0	<0.0001	55.56	5.24	0.0001	154.55	25.71	>0.05	100.00	0.00	>0.05	87.50	17.68	>0.05
3A	88.89	0	>0.05	40.74	5.24	<0.0001	136.36	38.57	>0.05	100.00	0.00	>0.05	50.00	0.00	<0.0001
4A	144.44	15.71	0.0058	88.89	10.48	>0.05	72.73	0.00	>0.05	100.00	0.00	>0.05	68.75	44.19	0.0311
5A	177.78	0	<0.0001	92.59	5.24	>0.05	127.27	25.71	>0.05	87.50	17.68	>0.05	25.00	0.00	<0.0001
6A	111.11	31.43	>0.05	85.19	15.71	>0.05	163.64	25.71	0.0134	100.00	0.00	>0.05	62.50	17.68	0.0046
7A	88.89	0	>0.05	70.37	15.71	0.0261	127.27	25.71	>0.05	100.00	0.00	>0.05	50.00	0.00	<0.0001
8A	77.78	15.71	>0.05	74.07	20.95	>0.05	163.64	25.71	0.0134	87.50	17.68	>0.05	75.00	17.68	>0.05
9A	144.44	15.71	0.0058	81.48	0.00	>0.05	122.73	19.28	>0.05	87.50	17.68	>0.05	50.00	0.00	<0.0001
10A	0	0	<0.0001	85.19	15.71	>0.05	163.64	25.71	0.0134	25.00	0.00	<0.0001	87.50	17.68	>0.05
11A	0	0	<0.0001	92.59	15.71	>0.05	209.09	12.86	<0.0001	50.00	0.00	<0.0001	75.00	17.68	>0.05
12A	0	0	<0.0001	114.81	5.24	>0.05	190.91	12.86	<0.0001	25.00	0.00	<0.0001	50.00	0.00	<0.0001
13A	88.89	0	>0.05	96.30	10.48	>0.05	54.55	0.00	>0.05	87.50	17.68	>0.05	50.00	0.00	<0.0001
14A	77.78	15.71	>0.05	81.48	0.00	>0.05	86.36	19.28	>0.05	125.00	0.00	0.0435	50.00	0.00	<0.0001
1B	0	0	<0.0001	48.15	5.24	<0.0001	150.00	19.28	>0.05	100.00	0.00	>0.05	43.75	8.84	<0.0001
2B	155.56	31.43	0.0002	44.44	0.00	<0.0001	136.36	38.57	>0.05	100.00	0.00	>0.05	50.00	0.00	<0.0001
3B	88.89	0	>0.05	48.15	5.24	<0.0001	200.00	25.71	<0.0001	125.00	0.00	0.0435	43.75	8.84	<0.0001
4B	133.33	31.43	0.0823	88.89	10.48	>0.05	163.64	25.71	0.0134	75.00	0.00	0.0435	50.00	0.00	<0.0001
5B	155.56	31.43	0.0002	81.48	10.48	>0.05	181.82	0.00	0.0005	87.50	17.68	>0.05	25.00	0.00	<0.0001
6B	88.89	0	>0.05	74.07	10.48	0.0765	150.00	19.28	>0.05	87.50	17.68	>0.05	25.00	0.00	<0.0001
7B	55.56	15.73	0.0058	62.96	26.19	0.0021	127.27	25.71	>0.05	100.00	0.00	>0.05	50.00	0.00	<0.0001
8B	88.89	0	>0.05	59.26	10.48	0.0005	145.45	0.00	>0.05	75.00	0.00	0.0435	50.00	0.00	<0.0001
9B	77.78	15.71	>0.05	66.67	0.00	0.0079	163.64	25.71	0.0134	100.00	0.00	>0.05	75.00	17.68	>0.05
10B	0	0	<0.0001	92.59	5.24	>0.05	181.82	25.71	0.0005	25.00	0.00	<0.0001	50.00	0.00	<0.0001
11B	0	0	<0.0001	100.00	5.24	>0.05	190.91	38.57	<0.0001	25.00	0.00	<0.0001	50.00	0.00	<0.0001
12B	0	0	<0.0001	103.70	10.48	>0.05	177.27	6.43	0.0012	25.00	0.00	<0.0001	50.00	0.00	<0.0001
13B	88.89	0	>0.05	92.59	15.71	>0.05	63.64	12.86	>0.05	87.50	17.68	>0.05	50.00	0.00	<0.0001
14B	55.56	15.71	0.0058	81.48	10.48	>0.05	72.73	0.00	>0.05	112.50	17.68	>0.05	50.00	0.00	<0.0001
DMSO	88.89	0	>0.05	62.96	5.24	0.0021	81.82	12.86	>0.05	87.50	17.68	>0.05	81.25	8.84	>0.05

Series A (*para*-SO_2_NH_2_) tends to be more effective in inhibiting haemolysin, while Series B (*meta*-SO_2_NH_2_) more effectively inhibits lecithinase, critical factors in tissue necrosis and inflammation. However, most compounds stimulate gelatinase, which can increase invasiveness. All things considered, series A provides a more favourable profile; nonetheless, it necessitates the use of substances that do not elevate gelatinase (3A, 13A, 14A).

In the case of the *C. albicans* strain (Table 4), haemolysin activity was partially reduced (yellow, 62.5%–87.5%) by series A and Series B compounds, The most significant inhibition was exhibited by the compounds 1A, 1B, and 10A (p < 0.05). Reducing haemolysin limits iron access and decreases tissue destruction, an advantage in the control of chronic infections (Cassat and Skaar, 2013). Any decrease in the activity of these enzymes reduces local necrosis and inflammation (Gomes et al., 2017). The production of organic acids was completely inhibited by the compounds 7A-B (*p*-^t^Bu-phenyl) and 9A-B (*m, p*-diOCH3-phenyl) without interfering with the basic structure, while for the compounds 8A and 1B, the activity was influenced by the basic structure. In the case of couple 1A-B, 1A led to the stimulation of acidic environment production (162.50 ± 17.68, p < 0.001). Acidification of the environment is a classic mechanism by which *C. albicans* facilitates its invasion and biofilm stability (Cauchie et al., 2017). A more acidic microenvironment affects host cells and delays tissue regeneration, contributes to biofilm tolerance, and can reduce the effectiveness of some antifungals (Pan et al., 2023). In the case of aesculin hydrolase, most compounds stimulated secretion (red, >100%), with a few exceptions (1A, 8A, 11A, 13A, and 10B). Among these compounds, only 11A significantly reduced enzymatic activity (p < 0.05). Higher glycohydrolase activity leads to increased metabolic adaptability both in the biofilm and in the tissue (Crabbé et al., 2019). Most of the tested compounds reduce lipase activity (yellow, 20.83%–66.67%), which is beneficial. A single compound led to stimulation, but with statistically insignificant values (116.67 ± 23.57, p > 0.05). Series A tended to reduce lipase more frequently (e.g., 1-4A, 7A, 9-14A), while in series B, there were more values close to the strain control. Reduced synthesis of lipase diminishes invasion and helps preserve the host’s lipid barrier. All things considered, Series A offers a more advantageous profile for decreasing *C. albicans* virulence, especially by restricting acidification and lipases activity. The strain control was negative for DNase, lecithinase, and gelatinase.

**TABLE 4:** Effect of sulfonamide derivatives on the secretion of enzymatic virulence factors (*C. albicans*). The colours used indicate: green – complete inhibition; yellow – reduced enzymatic activity; orange – activity similar to the strain control; red – stimulation of enzymatic activity. Statistical analysis was performed using Brown-Forsythe and Welch ANOVA test followed by Dunnett’s multiple comparisons test (n = 3).

Compound/Reference tested strain	Hemolysin			Organic acidity/Alkalinity			Aesculin hydrolyse			Lipase		
	Mean	Std. dev.	p-value	Mean	Std. dev.	p-value	Mean	Std. dev.	p-value	Mean	Std. dev.	p-value
*C. albicans*	100	0	-	100	0	-	100	0	-	100	0	-
1A	62.5	17.68	0.0160	162.50	17.68	0.0002	95.00	10.61	>0.05	58.33	11.79	0.0012
2A	75	17.68	>0.05	100.00	0.00	>0.05	115.00	7.07	>0.05	66.67	0.00	0.0178
3A	100	0	>0.05	62.50	17.68	0.0708	115.00	7.07	>0.05	66.67	23.57	0.0178
4A	100	17.68	>0.05	150.00	35.36	0.0045	110.00	0.00	>0.05	66.67	0.00	0.0178
5A	87.5	17.68	>0.05	162.50	17.68	0.0002	120.00	0.00	0.0185	91.67	11.79	>0.05
6A	87.5	17.68	>0.05	187.50	17.68	<0.0001	125.00	7.07	0.0013	83.33	0.00	>0.05
7A	75	0	>0.05	0.00	0.00	<0.0001	125.00	7.07	0.0013	66.67	0.00	0.0178
8A	87.5	17.68	>0.05	0.00	0.00	<0.0001	97.50	3.54	>0.05	100.00	23.57	>0.05
9A	75	0	>0.05	0.00	0.00	<0.0001	125.00	7.07	0.0013	62.50	17.68	0.0049
10A	62.5	17.68	0.0160	100.00	0.00	>0.05	100.00	0.00	>0.05	25.00	11.79	<0.0001
11A	75	0	>0.05	100.00	0.00	>0.05	80.00	14.14	0.0185	41.67	11.79	<0.0001
12A	87.5	17.68	>0.05	100.00	0.00	>0.05	105.00	7.07	>0.05	20.83	5.89	<0.0001
13A	100	0	>0.05	112.50	17.68	>0.05	95.00	7.07	>0.05	66.67	0.00	0.0178
14A	100	0	>0.05	112.50	17.68	>0.05	115.00	7.07	>0.05	33.33	0.00	<0.0001
1B	62.5	17.68	0.0160	0.00	0.00	<0.0001	105.00	7.07	>0.05	66.67	0.00	0.0178
2B	75	0	>0.05	200.00	0.00	<0.0001	120.00	10.61	0.0185	66.67	23.57	0.0178
3B	87.5	17.68	>0.05	50.00	0.00	0.0045	115.00	7.07	>0.05	66.67	0.00	0.0178
4B	75	0	>0.05	100.00	0.00	>0.05	120.00	0.00	0.0185	116.67	23.57	>0.05
5B	87.5	17.68	>0.05	362.50	17.68	<0.0001	115.00	7.07	>0.05	91.67	11.79	>0.05
6B	75	0	>0.05	262.50	17.68	<0.0001	130.00	0.00	<0.0001	91.67	11.79	>0.05
7B	100	0	>0.05	0.00	0.00	<0.0001	115.00	7.07	>0.05	50.00	11.79	<0.0001
8B	100	8.84	>0.05	87.50	17.68	>0.05	110.00	0.00	>0.05	75.00	11.79	>0.05
9B	87.5	17.68	>0.05	0.00	0.00	<0.0001	115.00	7.07	>0.05	66.67	0.00	0.0178
10B	87.5	17.68	>0.05	125.00	35.36	>0.05	95.00	7.07	>0.05	25.00	11.79	<0.0001
11B	100	0	>0.05	62.50	17.68	0.0708	105.00	7.07	>0.05	41.67	11.79	<0.0001
12B	87.5	17.68	>0.05	100.00	0.00	>0.05	100.00	0.00	>0.05	58.33	11.79	0.0012
13B	100	0	>0.05	100.00	0.00	>0.05	105.00	7.07	>0.05	66.67	0.00	0.0178
14B	87.5	17.68	>0.05	175.00	35.36	<0.0001	110.00	14.14	>0.05	66.67	0.00	0.0178
DMSO	100	17.68	>0.05	87.50	17.68	>0.05	105.00	7.07	>0.05	100.0	0.00	>0.05

Overall, sulfonamide derivatives selectively modulate virulence of the tested Gram-positive and Gram-negative bacteria as well as the fungal strain, the anti-virulence effect being also influenced by the *para*-SO_2_NH_2_ (series A) vs. *meta*-SO_2_NH_2_ (series B) positioning. Overall, Series A (*para*) offers the most favourable anti-virulence profile: it produces robust and consistent inhibition of haemolysin in *S. aureus* and frequently reduces aesculin hydrolase, tends to diminish lipase in *C. albicans*, and includes compounds that limit acidification. In *P. aeruginosa*, it maintains strong lecithinase inhibition and, for a subset of compounds, reduces lipase activity. The B series (*meta*) remains active, but it exhibits also undesirable increases in certain factors (more frequent stimulation of acid production in *C. albicans* and of hemolysin/gelatinase in *P. aeruginosa*), which makes it less consistent as a virulence modulator. Statistical analysis utilizing the Brown-Forsythe ANOVA followed by Dunnett’s test verified that the observed decreases in virulence factor expression were significant, indicating that the inhibitory patterns across the investigated drugs were consistent.

Among the analysed derivatives, the most promising compounds proved to be 14B *for S. aureus*, 13B for *P. aeruginosa*, and 8A for *C. albicans*, each successfully inhibiting the major virulence factors relevant to the respective species, i.e., haemolysin, lecithinase, and lipase, without undesirable stimulation of gelatinase or excessive acid production, which proves their potential for application in the therapy of chronic polymicrobial infections.

The antimicrobial evaluation of the synthesized sulfonamide derivatives, grouped into A and B series, reveals clear structure–activity relationships (SARs) and highlights the dual potential of these compounds as both carbonic anhydrase (CA) inhibitors (Al-Matarneh et al., 2024; Al-Matarneh et al., 2025) and antimicrobial agents. These effects can be rationalized by examining the substitution patterns, electronic properties of the R groups, and the known affinity of sulfonamides toward carbonic anhydrase isoforms—particularly human CA I and II and analogue microbial β- and γ-class CAs.

Compounds from the B series, bearing the sulfonamide group in the *meta* position, demonstrated superior antibacterial activity against *S. aureus* and *P. aeruginosa*. This was particularly evident in compounds such as 9B, 4B, 5B, and 6B, which exhibited some of the lowest MIC and IC50 values. Although *meta*-substitution leads to a misaligned geometry for effective binding to the zinc ion in human CA I and II, this orientation appears favorable for targeting microbial CAs, particularly the β- and γ-class isoforms present in bacteria. This suggests that the enhanced antibacterial effects of 3-SA compounds are likely attributed to selective inhibition of microbial CAs, rather than host CA I/II activity. Additionally, the presence of electron-withdrawing groups (EWGs) such as–CN, –CF_3_, and–Br further increased antimicrobial potency by enhancing compound polarity, facilitating bacterial cell penetration, or improving binding to microbial CA active sites.

In contrast, compounds in the 4-SA series, with *para*-sulfonamide substitution, showed weaker antibacterial but stronger antifungal activity, particularly against *C. albicans*. Compounds such as 2A, 7A, and 8A stood out due to their low MICs and strong IC_50_ values. *Para*-substituted sulfonamides are known to align optimally with the zinc-binding site of human CA II, and since fungal carbonic anhydrases—such as *C. albicans* Nce103 (a β-class CA)—share structural and mechanistic features with human CA II, this may explain the superior antifungal performance of the 4-SA series. The increased activity in this case could arise from dual inhibition of fungal CA and secondary cellular targets. Additionally, moderately polar and electron-donating groups (e.g., –OH, –OCH_3_) in 2A and 8A may enhance hydrogen bonding or π-π stacking interactions within fungal CA active sites or membrane environments, contributing to efficacy.

The IC_50_ and MIC data also align with literature findings showing that *para*-substituted sulfonamides are generally more effective human CA I/II inhibitors, while *meta*-substituted analogs are less potent toward these isoforms but may offer microbial CA selectivity. This distinction is critical: while inhibition of human CA I/II may risk host toxicity, the high activity of *meta*-substituted (3-SA) compounds against bacteria without strong human CA I/II inhibition suggests a favorable selectivity profile and potential for antimicrobial development. Importantly, the expression of *β*- and *γ*-class CAs in *S. aureus* and *P. aeruginosa*, and the *β*-class CA Nce103 in *C. albicans*, underscores the vital role of microbial CAs in CO_2_ hydration, pH regulation, and virulence. Inhibiting these enzymes leads to physiological disruption in the pathogens, reducing their survival and infectivity. The structural variations within the 3-SA and 4-SA series (Figure 5), specifically the position of the sulfonamide moiety and the electronic nature of the R substituent, directly influence binding affinity and antimicrobial outcomes.

### 2.3 Hemolytic activity assessment

**FIGURE 6:**
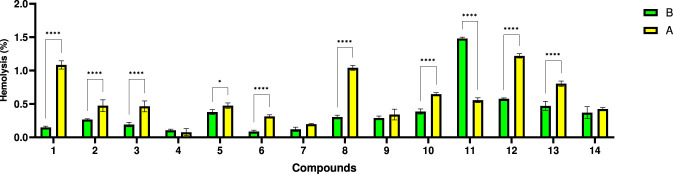
Comparative hemolysis of erythrocyte suspension by (A and b sulfonamide derivatives series at 1 mg/mL. Statistical analysis was performed using the two-way ANOVA method (Sidak’s multiple comparisons test), and significance was expressed as follows: *p < 0.05, ****p < 0.0001.

Investigations regarding hemocompatibility are essential in the development of therapeutic agents intended for the healing/improvement of chronic wound symptoms because they ensure an adequate safety profile and help in understanding their mechanism of action. This assay is essential for avoiding side effects and optimizing formulations to promote healing with minimal damage thus ensuring their usability in open wounds (Jonkman et al., 2014; Norahan et al., 2023). The results of tested compounds regarding their hemolytic effect are presented in Figure 6.

No compound was found to be hemolytic at 1 mg/mL, with hemolysis being below 5% (Figure 5). Hemolysis decreased in order 11A > 12A > 13A > 10A > 5A > 14A > 8A > 9A > 2A > 3A > 1A > 7A > 4A > 6A for series 4-SA, and for series 3-SA in the order 12B > 1B > 8B > 13B > 10B > 11B > 5B > 2B > 3B > 14B > 9B > 6B > 7B > 4B. The 3-SA series of compounds proved to be significantly more hemolytic than the 4-SA series, except for the pair 11A/11B. Compounds 11A, 12A, 12B, 8B exhibited the highest hemolytic index, likely due to the presence of multiple phenolic/methoxy groups, which may enhance interaction with the erythrocyte membrane through hydrogen bonding and local destabilization. Compounds 7B, 4B, 4A, and 6A contain substituents that reduce hemolytic activity, likely due to the lack of strong interactions with the phospholipids of the erythrocyte membrane (Greco et al., 2020). Compounds with methoxy substituents or other hydrophobic groups can destabilize the membrane through direct insertion. Hydrophilic groups can prevent excessive interaction with the cell membrane and reduce hemolysis (Jeswani et al., 2015).

## 3 Biocompatibility

**FIGURE 7:**
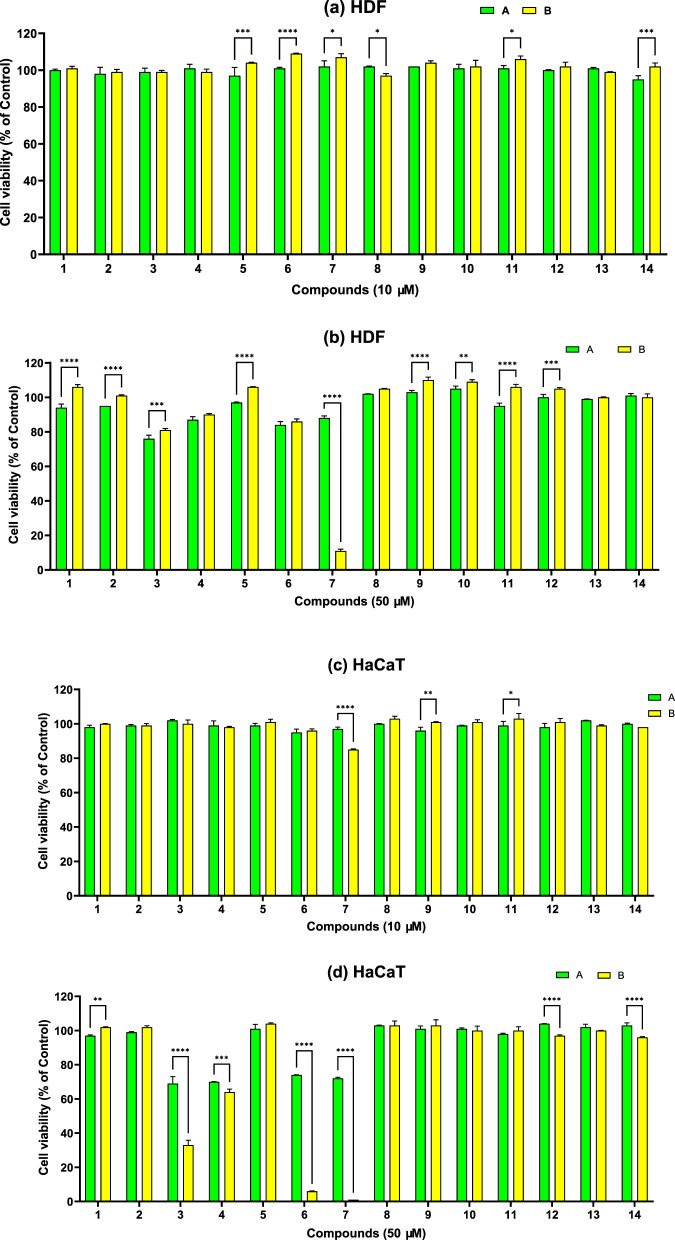
Comparative biocompatibility of A and B sulfonamide derivatives series on: **(a)** Dermal fibroblasts (HDF) at 10 μM; **(b)** Dermal fibroblasts at 50 μM; **(c)** Keratinocytes (HaCaT) at 10 μM; **(d)** Keratinocytes at 50 µM. Statistical analysis was performed using the two-way ANOVA method (Sidak’s multiple comparisons test), and significance was expressed as follows: *p < 0.05, **p < 0.01, ***p < 0.001, ****p < 0.0001.

For wound healing and dermal antimicrobial applications, biocompatibility assessment on dermal cells is crucial. The results of biocompatibility testing of compounds on normal dermal fibroblasts and keratinocytes are presented in Figure 7. All compounds were biocompatible at 10 µM concentration on both dermal fibroblasts and keratinocytes. At 50 μM, some compounds induced a slight decrease in fibroblasts (3A- 76%) and keratinocytes (3A- 69%, 4A–70%, 6A–74%, 7A–72%, 4B–64%) viability, near the cytotoxic threshold (70%). However, at 50 μM, compounds 3B, 6B and 7B were highly cytotoxic for fibroblasts (7B- 11%) and keratinocytes (3B- 33%, 6B–6%, 7B–1%). Interestingly, a higher cytotoxicity at 50 µM was observed for compounds of the 3-SA series: 7B vs. 7A on dermal fibroblasts (Figure 7b), 6B vs. 6A, 7B vs. 7A and 3B vs. 3A.

**FIGURE 8:**
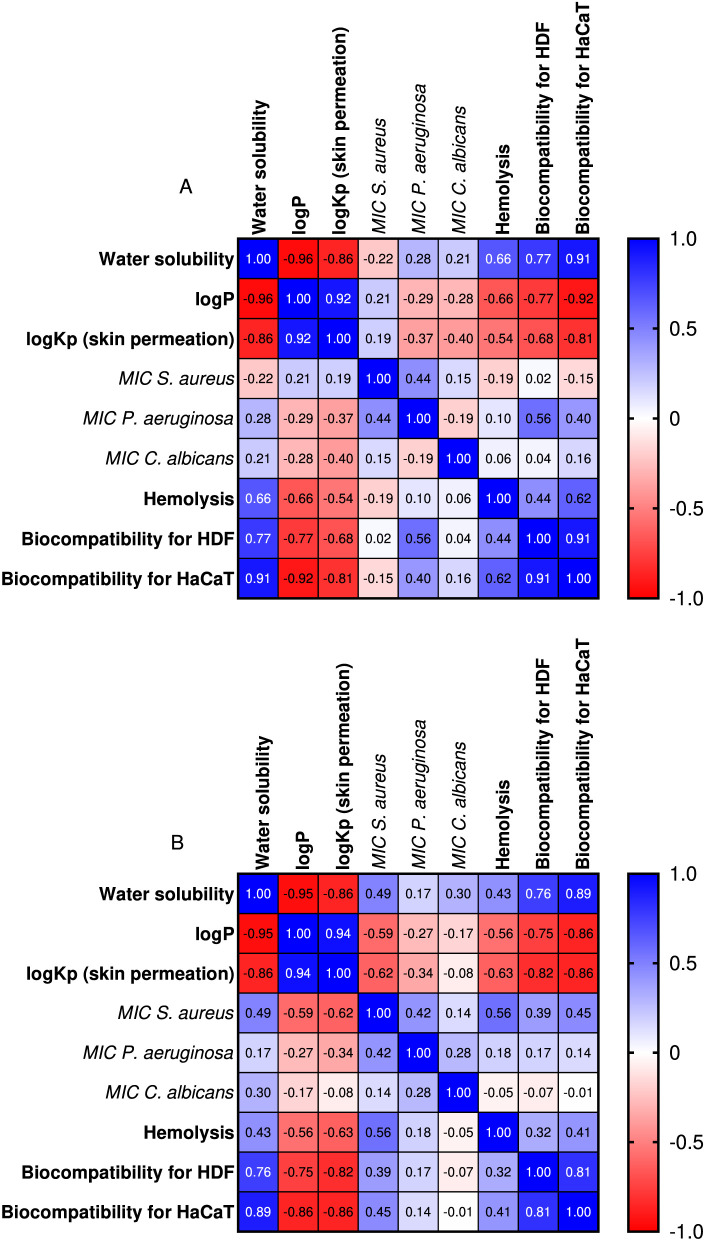
The Pearson correlation between physico-chemical parameters, antimicrobial activity and biocompatibility for HaCaT cell line, HDF cell line and erythrocytes for **(A,B)** sulfonamide derivatives series.

Several compounds which exhibited the highest activity against *S. aureus* (3A, 6A, 4B, 5B, 11B, 12B, 13B and 14B), *P. aeruginosa* (4B and 5B) and *C. albicans* (2A, 3A, 4A, 5A, 6A, 7A, 11A, 12A, 13A, 14A, 4B, 5B, 9B and 11B) also exhibited good biocompatibility on dermal fibroblasts and keratinocytes, even at higher concentrations (50 µM). However, while compounds 6B and 7B were effective on *P. aeruginosa* and *C. albicans* and compound 3B was effective on *S. aureus* and *C. albicans*, their marked cytotoxicity at 50 µM in dermal keratinocytes and fibroblasts suggests caution in future *in vivo* studies.

The Pearson correlation for the compounds in series A (Figure 8A) showed that as lipophilicity increases, aqueous solubility decreases (solubility vs. logP: r = −0.96). Also, the more lipophilic the compound, the greater its skin permeability (logKp vs. logP: r = +0.92). These effects correlate very well and are in line with the Potts–Guy type relationships (log Kp increases with logP and decreases with molecular weight) (Burli et al., 2020). The strong negative correlations between cell viability (HDF/HaCaT) and logP/logKp (HDF: -0.77/-0.68; HaCaT: -0.92/-0.81) indicate that the more lipophilic/permeable the compounds are, the lower their biocompatibility is (likely due to membrane interactions/intracellular accumulation). Compounds that are more soluble in water appear to be more biocompatible for skin cells, with viability (HDF/HaCaT) vs. solubility having a positive correlation (HDF: +0.77; HaCaT: +0.91). Correlating haemolysis with solubility (r = +0.66) and haemolysis with logP/logKp (r = −0.66/-0.54) produced moderate effects. The more soluble chemicals seem more haemolytic at the studied concentration, and as lipophilicity rises, so does hemocompatibility. Erythrocyte exposure can be reduced by limiting lipophilic substances by protein binding or solubility/precipitation. More soluble compounds can elicit osmotic/surfactant-like lysis and more readily reach effective concentrations.

Antimicrobial potency correlated less strongly with physico-chemical properties. In the case of the MIC values obtained against the *S. aureus* strain, weak correlations were observed (solubility −0.22; logP +0.21; logKp +0.19), and the flattened SAR indicates that potency was not directly dictated by these three parameters. The correlation between MIC values against *P. aeruginosa* and solubility (+0.28), logP (−0.29), and logKp (−0.37) indicates a tendency for more lipophilic/permeable compounds to have better antimicrobial activity, likely due to the outer membrane composed of lipopolysaccharides, which is specific to Gram-negative bacteria (an external barrier). For *C. albicans*, MIC values showed weak associations with water solubility (r = 0.21) and lipophilicity (logP, r = −0.28), and a weak to moderate inverse correlation with predicted skin permeation (logKp, r = −0.40). None of them reached statistical significance at n = 14, indicating that the antifungal potency within this chemical series was not primarily determined by solubility or lipophilicity, but that permeation seems to play a modest role. The correlation between MIC values across species was moderate for *S. aureus* vs. *P. aeruginosa* (r = 0.44), but very weak compared to *C. albicans*, highlighting species specificity.

Weak correlations between *S. aureus* MIC values vs. viability (HDF: 0.02; HaCaT: −0.15) and *S. aureus* MIC values vs. hemolysis (−0.19) are close to zero, suggesting that efficacy was not reflected in toxicity, which is good for SAR optimization. The same effects were observed in the case of the *C. albicans* strain. While the MIC values for *P. aeruginosa* vs. haemolysis (0.10) were near zero, indicating that efficacy was not reflected in hemocompatibility, the effects between the MIC values for *P. aeruginosa* vs. viability (HDF: 0.56; HaCaT: 0.40) were moderate, emphasising that the lower the MIC, the better the biocompatibility. This is likely because all compounds were found to be hemocompatible at the tested concentration.

The Pearson correlation for the compounds in series B (Figure 8B) showed that as lipophilicity increases, aqueous solubility decreases (solubility vs. logP: r = −0.95). Also, the more lipophilic the compound is, the greater its skin permeability (logKp vs. logP: r = +0.94). These effects correlate well and are in line with the Potts–Guy type relationships (log Kp increases with logP and decreases with molecular weight) (Burli et al., 2020). Biocompatibility (HDF/HaCaT) correlates positively with solubility (+0.76/+0.89) and negatively with logP (−0.75/−0.86) and logKp (−0.82/−0.86), thus as compounds become more lipophilic/permeable, viability decreases. These effects correlate with the specialized literature in that *in vitro* toxicity often increases with a high logP due to membrane interactions/intracellular accumulation (Komleva et al., 2015). Hemolysis was moderately positive with solubility (+0.43) and negative with logP/logKp (−0.56/−0.63), likely because highly lipophilic analogs can precipitate or bind to proteins, resulting in a lower hemolytic signal.

The correlation between the MIC values against *P. aeruginosa* and the physico-chemical parameters was relatively weak (MIC vs. logP: −0.27; MIC vs. logKp: −0.34; MIC vs. solubility: +0.17), indicating a tendency for more lipophilic/permeable compounds to have lower MIC values (better potency), a similar effect to that observed for the compounds in series A. In the case of the *S. aureus* strain, the correlation with physicochemical parameters was moderate (MIC vs. solubility: 0.49, MIC vs. logP: -0.59, MIC vs. logKp: -0.62), with the observation that increasing lipophilicity facilitates membrane interaction and passive diffusion, and a higher logKp went in the same direction. Conversely, highly soluble (and therefore less lipophilic) analogues may have difficulty penetrating the membrane, resulting in slightly higher MIC values. For the activity of the compounds against *C. albicans*, the correlation with logP (−0.17) and logKp (−0.08) was very weak, while the correlation with solubility was weak to moderate (0.30). In the case of the 14 analogs, the MIC against *S. aureus* was positively correlated with hemolysis (r = 0.56), indicating that more potent compounds (lower MIC) tend to be less hemolytic.

Overall, in both series, the Potts–Guy type relationships are confirmed (water solubility decreases with increasing logP, and logKp increases with logP), biocompatibility (HDF/HaCaT) decreases with lipophilicity/permeability and increases with solubility, haemolysis was higher for more soluble compounds, and among the notable differences, it was highlighted that only in series B was the potency against *S. aureus* moderately correlated with logP/logKp, while the trend against *P. aeruginosa* was weak in both series, the relationships were negligible against *C. albicans*, and the positive correlation of MIC values against *S. aureus* and haemolysis (r = 0.56) indicated a favourable agreement between antimicrobial potency and hemocompatibility, opening the possibility of identifying selective candidates against microbial cells.

General correlations were observed between lipophilicity, solubility, and antimicrobial activity, but certain derivatives deviated from these trends. These deviations can be explained by the specific effects of the substituents (electronic, steric) and by the differences in the molecular backbone between the 4SA and 3SA series, which non-linearly influence solubility, permeability, and interactions with the microbial cell membrane. Derivatives bearing bulky or electron-withdrawing substituents (e.g., halogens, CF_3_, or benzo[d] (Almalki et al., 2022; Krátký, 2024) dioxole rings) can exhibit altered interactions with the membrane or solvation profiles, which can affect both antimicrobial potency and biocompatibility. Additionally, the differences between series A (4SA) and B (3SA) can arise from the change in the sulfonamide linkage position and the presence of the nitro substituent in series B, which alters the overall dipole moment and intermolecular interactions, influencing permeability and target binding in a non-linear manner. Therefore, while the overall trends remain consistent, local deviations are in line with the specific substituent and positional effects within each structure.

### 3.1 Study limitations

This manuscript was intentionally designed as a comparative, early-stage screen to prioritize chemotypes (*para*-vs. *meta*-sulfonamide across 14 substituents) with antimicrobial, antibiofilm and anti-virulence potential. Several boundaries of scope follow from this design: i) our experiments were performed on ATCC reference strains under static, polystyrene plate conditions. The antibiofilm assays targeted early adhesion/initial biomass, the MBEC definition relying on a biomass surrogate (crystal violet, ≥95% reduction) rather than viability-specific readouts. Clinical isolates and polymicrobial models were not included; ii) MIC/MMC/MBEC were assessed at single incubation time points; time-kill, regrowth/post-antibiotic effect, and long-term biofilm viability (e.g., CFU after dispersal, live/dead confocal imaging) were not performed; iii) while the SAR trends are compatible with CA–related effects and anti-virulence activity at sub-inhibitory concentrations, the study did not include direct enzyme assays with purified microbial vs. human CAs (no Ki/IC_50_ or selectivity indices) nor molecular target-engagement data. Likewise, the virulence-factor reductions were not linked to specific regulatory pathways; iv) solubility and lipophilicity were predicted *in silico*; v) biocompatibility/hemocompatibility were screened at a single concentration to compare the two series. Full dose–response cytotoxicity (IC_50_) and therapeutic index calculations were not completed for all compounds within this manuscript (performed only for a subset in subsequent work). No *ex vivo* skin or *in vivo* tolerability/irritation tests were included; vi) gentamicin (bacteria) and ketoconazole (*C. albicans*) were added as reference standards to contextualize activity; however, the study was not powered or designed to establish non-inferiority/superiority *versus* standard-of-care agents, vii) the propensity for resistance development was not assessed and synergy/antagonism with antibiotics commonly used in wound care was not evaluated; viii) although variance homogeneity was verified and robust ANOVA procedures were used when normality was violated, the sample sizes typical of screening studies limit the precision of some estimates and the generalizability across strain diversity; ix) A few; deeper structure–property analyses (including experimental ADME and membrane interaction studies) will be needed to rationalize the exceptions deviated from the dominant SAR trends.

## 4 Conclusion

This study evaluated two series of pyrrol-2-one sulfonamide derivatives, differing in the sulfonamide position (*para*, 4-SA; *meta*, 3-SA) and substituted with 14 distinct electron-donating or electron-withdrawing R groups, for their antimicrobial and anti-virulence potential. *Meta*-substituted derivatives (Series B) exhibited stronger antibacterial activity, likely via selective inhibition of microbial β-/γ-class carbonic anhydrases, whereas *para*-substituted derivatives (Series A) demonstrated superior antifungal activity, antibiofilm effects, and inhibition of key virulence factors, including haemolysin, lipase, and lecithinase. Structure–activity relationship analysis revealed that *para*-substitution aligns with human CA II, enhancing antifungal efficacy, while *meta*-substitution favors microbial CA targeting, explaining antibacterial selectivity. Physicochemical properties influenced biocompatibility: increased lipophilicity improved skin permeability but reduced solubility and cellular viability, whereas haemolysis was higher for more soluble compounds. Importantly, none of the compounds showed haemolytic activity at 1 mg/mL, and all were well tolerated by human dermal fibroblasts and keratinocytes at 10 µM. The most promising derivatives—9B for *S. aureus* and *P. aeruginosa*, and 9A for *C. albicans*—consistently inhibited key virulence factors and represent promising candidates for adjuvant therapy in chronic polymicrobial infections. These findings underscore the critical influence of sulfonamide positioning and R-group electronics on antimicrobial potency, virulence modulation, and CA isoform selectivity, supporting the rational design of safe, pathogen-specific therapeutics for dermal and systemic infections. Future work on the compounds selected in this screening assay will address the following aspects: (i) performing direct CA inhibition assays on human and microbial isoforms with selectivity indices; (ii) expanding to clinical and polymicrobial biofilm models, *ex vivo* skin and *in vivo* validation; (iii) incorporating kinetic bactericidal/biocidal studies, viability-specific biofilm endpoints, and resistance-selection experiments; (iv) generating experimental ADME/biophysics and skin-permeation data and optimizing topical formulations; and (v) completing dose–response safety datasets and therapeutic index calculations for the prioritized compounds. Together, these steps will strengthen translational relevance and help de-risk the most promising candidates for topical anti-infective development.

## 5 Experimental

### 5.1 Antimicrobial activity

#### 5.1.1 Microbial strains

The antimicrobial activity was done on reference microbial strains, belonging to Gram positive bacteria (*S. aureus* ATCC 25923), Gram negative (*P. aeruginosa* ATCC 27853) and yeast (*C. albicans* ATCC 10231). All determinations were performed in technical triplicates.

#### 5.1.2 Quantitative antimicrobial activity assay (MIC assay)

The quantitative analysis was carried out by the serial binary microdilutions (10–0.16 mg/mL) method in liquid medium (Trypton Soy Broth for bacteria and Sabouraud for *C. albicans)* according to CLSI (2023), in 96-well plates, using solvent control (DMSO), positive control (untreated microbial strains) and negative control (sterility conditions). Each well was inoculated with 10 µL of microbial suspension (0.5 McFarland, 1.5 × 10^8^ CFU/mL). After 20-24 h of incubation at 37 °C, the MIC was established both macroscopically, as the lowest concentration of active substance, capable of inhibiting the microbial culture development, respectively the turbidity of the culture medium, and spectrophotometrically, at 620 nm. For each sample, a blank was made including culture medium and each sample concentration. For the concentrations at which a linear increase in cell viability was evident, the IC50 was calculated (the concentration of sample that inhibits by 50% the microbial growth).

#### 5.1.3 Minimum microbicidal concentrations (MMC) assay

To determine the minimum microbicidal concentrations (MMC), 5 µL content from the wells of the MIC plate where no microbial growth was observed were spotted to solid media (Muller Hilton for bacteria strains and Sabouraud agar for yeast). The plates were incubated for 20-24 h at 37 °C. The last concentration where no colony has grown was considered the CMM.

#### 5.1.4 Microbial adherence capacity to the inert substratum

Microbial adhesion was assessed using the crystal violet microtiter assay of biofilm mass after fixation with 120 μL methanol and stained after drying with 120 μL crystal violet (0.1%), subsequent to the MIC assay. The adhering biomass was resuspended in 120 μL of 33% acetic acid and stained with crystal violet, then its absorbance was measured at 490 nm.

#### 5.1.5 The microbial enzymes and organic acid production

Microbial strains were treated with a subinhibitory concentration of each compound and DMSO solvent and then, each strain was grown in the presence of a sub-inhibitory concentration of the tested compounds and DMSO (0.313 mg/mL) for 24 h at 37 °C, and subsequently, standard 0.5 McFarland (1.5 × 10^8^ CFU/mL) suspensions were prepared in sterile 0.85% NaCl solution. After incubation, the production of six enzymatic virulence factors (pore-forming toxins: lecithinase, lipase, hemolysins; exoenzymes: gelatinase, DNase, aesculin hydrolase) and pH change were evaluated using specific culture media (Corbu et al., 2021; Corbu et al., 2023). A volume of 10 µL of the treated and untreated microbial suspensions was spotted on these culture media and then incubated at 37 °C for 24 h. The impact of the compounds on the metabolic profile was evaluated semi-quantitatively by measuring the ratio between the colony diameter (C) and the diameter of the specific culture medium modification around the colony (D) and then applying the following formula:

$$\text{Inhibition of enzymatic activity }\left(\%\right)=100-\frac{D2-C2}{D1-C1}\;⨯\;100,$$

where, C1 = Control strain colony diameter (mm), D1 = Control strain zone of halo diameter (mm), C2 = Treated strain colony diameter (mm), and D2 = Treated strain zone of halo diameter (mm).

### 5.2 Hemocompatibility

A haemolysis assay was conducted using sheep red blood cells (RBCs). To prevent clotting, 9 mL of blood was mixed with 1 mL of 10% citric acid dextrose. After centrifugation at 5,000 rpm for 10 min at 4 °C, the supernatant containing plasma was discarded, and the RBC pellet was washed three times and resuspended in phosphate-buffered saline (PBS, 0.1 M, pH 7.4). For the assay, 100 μL of 1 mg/mL samples in DMSO were mixed with 400 μL of RBC suspension, gently inverted, and incubated at 37 °C for 60 min. Positive and negative controls used 1% Tryton X-100 and PBS, respectively. Following incubation, samples were centrifuged at 5,000 rpm for 10 min at 4 °C, and the supernatant was transferred to 96-well plates. Absorbance at 540 nm was measured.

### 5.3 Biocompatibility assessment

Biocompatibility of compounds was assessed on human dermal fibroblasts (HDF) and HaCaT human keratinocytes (both from Cytion GmbH, Eppelheim, Germany) using the CellTiter-Glo® 2.0 Assay (Promega, Madison, WI USA), according to the manufacturer’s instructions. Cells were cultured in αMEM (HDF) or DMEM (HaCaT) medium with 10% fetal bovine serum and 1% antibiotic-antimycotic (from PAN‐Biotech GmbH, Aidenbach, Germany or Sigma-Aldrich, St. Louis, MO, USA - DMEM). Cells were seeded into 96-well opaque white tissue culture-treated plates (50000 cells/mL) and allowed to adhere overnight in complete cell culture medium. Cells were then incubated with compounds (10 or 50 µM) for 24h, then CellTiter-Glo® reagent was added and luminescence was recorded using a FLUOstar® Omega microplate reader (BMG LABTECH, Ortenberg, Germany). The experiments were carried out in triplicate, and the viability of treated cells was expressed as a percentage of the viability of control cells (untreated). Data were represented as means ± standard deviations.

### 5.4 Statistical analysis

Data were expressed as means ± SD determined by technical triplicate analysis. The statistical analysis was conducted using GraphPad Prism v10 (GraphPad Software, San Diego, CA, USA). Data were analyzed using ordinary two-way ANOVA with Tukey’s multiple comparisons test, with a single pooled variance computed for comparison between derivative series and solvent used for IC50. The Brown–Forsythe ANOVA test, followed by the Dunnett test for multiple comparisons, was used to evaluate the inhibition of virulence factors, ensuring the robustness of the statistical analysis even when the data distribution did not strictly adhere to the normality assumption. An ordinary two-way ANOVA with Sidak’s multiple comparisons test, with a single pooled variance computed, was used for comparison between derivative series for the hemo-/biocompatibility. The level of significance was set to p < 0.05.

## Data Availability

The original contributions presented in the study are included in the article/supplementary material, further inquiries can be directed to the corresponding author.
